# Monitoring of population dynamics of *Corynebacterium glutamicum* by multiparameter flow cytometry

**DOI:** 10.1111/1751-7915.12018

**Published:** 2012-12-20

**Authors:** Andrea Neumeyer, Thomas Hübschmann, Susann Müller, Julia Frunzke

**Affiliations:** 1 Institute of Bio- and Geosciences, IBG-1: Biotechnology, Forschungszentrum Jülich52425, Jülich, Germany; 2 Department for Environmental Microbiology, Helmholtz-Centre for Environmental Research – UFZPermoserstr. 15, 04318, Leipzig, Germany

## Abstract

Phenotypic variation of microbial populations is a well-known phenomenon and may have significant impact on the success of industrial bioprocesses. Flow cytometry (FC) and the large repertoire of fluorescent dyes bring the high-throughput analysis of multiple parameters in single bacterial cells into reach. In this study, we evaluated a set of different fluorescent dyes for suitability in FC single cell analysis of the biotechnological platform organism *Corynebacterium glutamicum*. Already simple scattering properties of *C. glutamicum* cells in the flow cytometer were shown to provide valuable information on the growth activity of analysed cells. Furthermore, we used DAPI staining for a FC-based determination of the DNA content of *C. glutamicum* cells grown on standard minimal or complex media. Characteristic DNA patterns were observed mirroring the typical uncoupled DNA synthesis in the logarithmic (log) growth phase and are in agreement with a symmetric type of cell division of *C. glutamicum*. Application of the fluorescent dyes Syto 9, propidium iodide, and DiOC_2_(3) allowed the identification of subpopulations with reduced viability and membrane potential within early log and stationary phase populations. The presented data highlight the potential of FC-based analyses for online monitoring of *C. glutamicum* bioprocesses and provide a first reference for future applications and protocols.

## Introduction

It is a well-known fact that even isogenic microbial populations often exhibit significant variability at the single cell level, which consequently may impact the productivity and the economic success of a whole bioprocess (Lidstrom and Konopka, 2010). Conventional approaches for the analytical monitoring of biotechnological processes are, however, still dominated by bulk measurements providing average data for cellular fitness, productivity or physical parameters. Consequently, there is a lack in receiving statistically valid information about the complex phenotypic structure within populations of industrial microorganisms (Nebe-von-Caron *et al*., 2000; Müller and Nebe-von-Caron, 2010; da Silva *et al*., 2012).

In this context, flow cytometry (FC) represents a high resolution technique to assess phenotypic patterns of microbial populations and paves the way for novel high-throughput screening approaches in microbial strain development (Dietrich *et al*., 2010; Tracy *et al*., 2010). Based on the principle of hydrodynamic focusing, cells in a flow cytometer are transported in a fluid stream in a pearl chain-like arrangement. At the laser intercept cells passing the laser beam are analysed on the basis of their scattering (forward scatter, FSC, and side scatter, SSC) and fluorescence properties (dyes, natural fluorescence or recombinant proteins). Previous studies revealed that even simple parameters as the cell size, granularity or DNA content give already valuable insights into the metabolic status and the fitness of the whole population (Erlebach *et al*., 2000; Hewitt and Nebe-von-Caron, 2004; Müller, 2007). Membrane potential (MP) and integrity are further parameters, which can be analysed by the use of specific fluorescent dyes and do provide important information regarding the cellular stress response and viability parameters, which were shown in several studies to significantly correlate with productivity and economic efficiency of the bioprocess (Hewitt *et al*., 1999; David *et al*., 2011). When equipped with a cell sorting capability for separation of single bacterial cells, FC analyses can be complemented with further downstream investigations, such as microscopy, cultivation in liquid or on agar plates, or even *Omics*-based technologies (Bernas *et al*., 2006; Wiacek *et al*., 2006; Jehmlich *et al*., 2010).

The small cell size (1/1000 of the volume of a mammalian cell) and huge diversity with respect to cell wall composition and metabolism challenges FC approaches in the field of microbiology and affects measurement quality (Shapiro and Nebe-von-Caron, 2004; Müller and Nebe-von-Caron, 2010). Nowadays, a variety of fluorescent dyes is available to assess different phenotypic parameters, such as growth, MP or integrity. The majority of current protocols was, however, established and verified for only a small number of model species (Shapiro and Nebe-von-Caron, 2004). To end up with detailed and meaningful insights into the phenotypic structure of a species of interest, suitability of fluorescent dyes and staining protocols have to be adapted and optimized for every single bacterial species to be studied (Vives-Rego *et al*., 2000; Müller and Nebe-von-Caron, 2010).

Besides species like *Escherichia coli* or *Saccharomyces cerevisiae*, the Gram-positive soil bacterium *Corynebacterium glutamicum* represents one of the most important platform organisms in industrial biotechnology (Eggeling and Bott, 2005; Burkovski, 2008). Currently, *C. glutamicum* is used for the industrial production of more than 3 million tons of amino acids per year, mainly l-glutamate and l-lysine. Only few studies have yet applied single cell approaches for strain development or for the analysis of phenotypic variation of *C. glutamicum* populations (Frunzke *et al*., 2008; Binder *et al*., 2012; Grünberger *et al*., 2012; Mustafi *et al*., 2012). A study of our lab could show that even under standard cultivation conditions wild type populations of the strain *C. glutamicum* ATCC 13032 exhibit significant variation with respect to the activity of the large prophage CGP3 (Frunzke *et al*., 2008). FC was first used in two very recent studies for the development of biosensor-based high-throughput screening approaches for the isolation of metabolite producing mutants (Binder *et al*., 2012; Mustafi *et al*., 2012). In these studies the intracellular amino acid levels were monitored in single cells by using transcriptional regulators as natural sensor devices to turn on expression of a fluorescent protein in response to the internal amino acid concentration.

In this work, we used FC and cell sorting to analyse population dynamics of *C. glutamicum* with respect to cell size, granularity and DNA content at single cell resolution*.* Fluorescent dyes and staining protocols were established to assess membrane integrity and potential. Altogether, these data represent a first comprehensive insight into the phenotypic distribution of *C. glutamicum* populations and, thus, provide an important reference for future applications of FC as powerful tool for process monitoring and high-throughput screening for this important model organism.

## Results and discussion

### Size distribution of *C. glutamicum* wild type cells

Cells passing the laser beam in the flow chamber of a cytometer can be characterized on the basis of their scattering properties. It is a general assumption that the light scattering axial to the laser beam, so-called FSC, can be correlated with the size of a cell, whereas light scattering perpendicular to the laser beam (SSC) is indicative for the internal complexity or granularity of a cell (Müller and Nebe-von-Caron, 2010).

**Figure 1:**
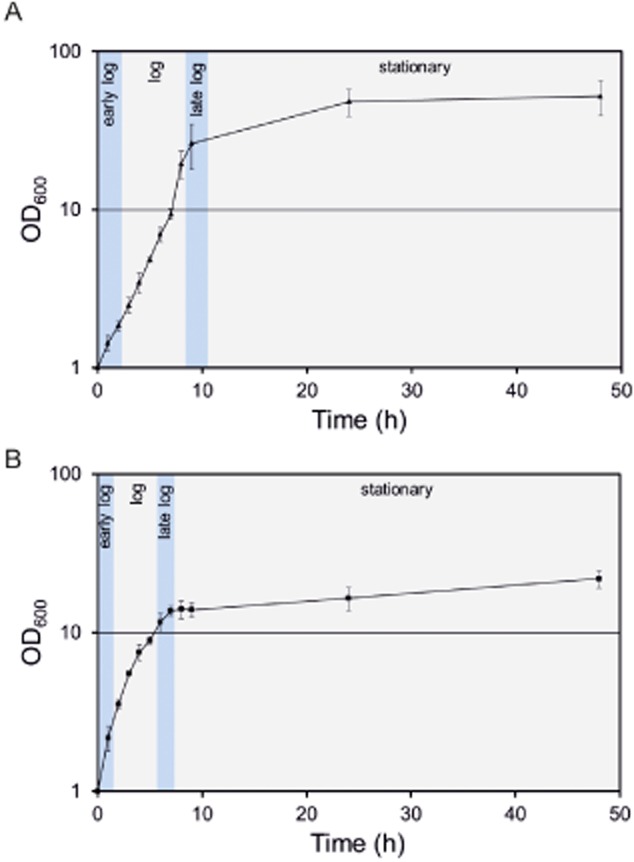
Growth of C. glutamicum ATCC 13032 on (A) CGXII minimal medium with 4% glucose and (B) BHI complex medium in a 50 ml shaking flask culture at 30°C and 120 r.p.m.

**Figure 2:**
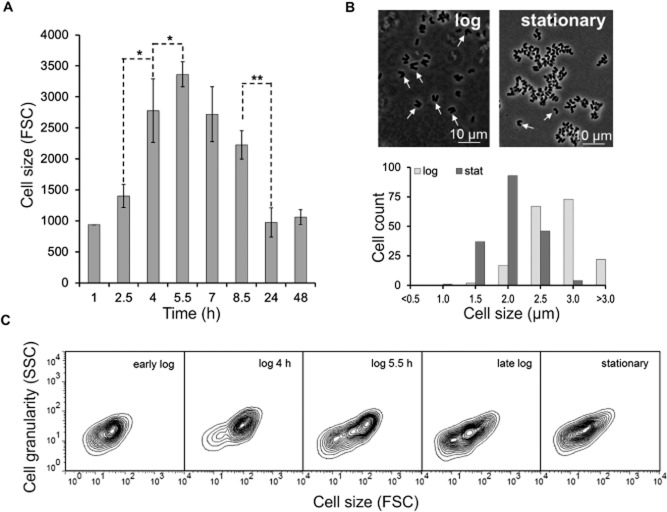
Size distribution of *C. glutamicum* cells during growth on CGXII minimal medium.A. Mean in forward scatter (FSC). Bars represent the mean ± SD of three independent biological replicates. The asterisks depict significant differences between different time points (*P ≤ 0.05, **P ≤ 0.01).B. Microscopic analysis of *C. glutamicum* cells of the exponential (5.5 h after inoculation) and stationary growth phase (24 h).C. Contour plots of *C. glutamicum* cells (FSC versus SSC).

**Figure 3:**
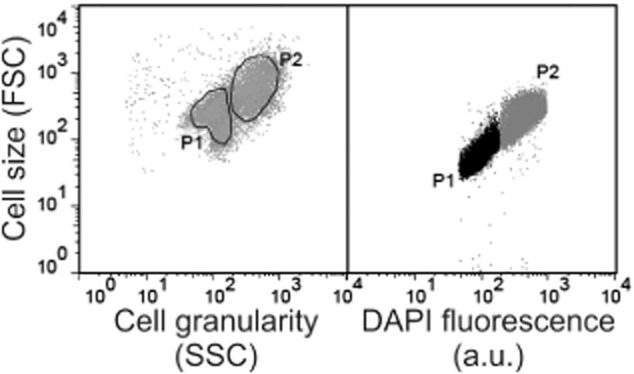
Correlation between cell size (FSC signal) and DAPI fluorescence. Gated populations (P1 and P2) are shown as dot plots: Cell size (FSC) against granularity (SSC) and FSC against DAPI fluorescence.

In the case of *C. glutamicum* ATCC 13032, we observed a characteristic FSC distribution during growth in CGXII minimal medium containing 4% glucose as carbon source (Fig. 1). Cells of the early logarithmic (log) or stationary phase exhibited the smallest FSC signal. This signal significantly increased during the mid-log phase and reached a maximum approximately 5.5 h after inoculation (Fig. 2A). Microscopic analysis confirmed these findings demonstrating a difference in cell size of log phase cells (5.5 h: 2.58 μm ± 0.31) and stationary cells (24 h: 1.66 μm ± 0.31) (Fig. 2B). In Fig. 2C the FSC versus SSC distribution is shown as contour plots highlighting the formation of a second subpopulation in log phase populations. In order to verify whether the high-FSC/SSC subpopulation in fact represents cells in a proliferating state we used DAPI (4′,6-diamidino-2′-phenylindole) staining to correlate scattering properties with the DNA content of the respective populations. The fluorescent dye DAPI is routinely used for DNA quantification of bacterial and eukaryotic cells and binds to A/T-rich regions of DNA resulting in a bright and stable fluorescence signal. Due to its low emission maximum (463 nm) DAPI can also nicely be combined with other stains. As expected, cells with a low-FSC signal (P1) also showed a lower DAPI fluorescence than high-FSC cells (P2) (Fig. 3).

When interpreting cell size distributions, the unique and multilayered cell wall structure of *C. glutamicum* has to be considered (Eggeling *et al*., 2008). A characteristic feature of the group of mycolata, including the genera *Corynebacterium*, *Mycobacterium* and *Nocardia*, is the formation of so-called V-shaped cells caused by a snapping cell division (see arrows in Fig. 2B). The cell wall of the mycolata is surrounded by a lipid-rich envelope, regarded as outer membrane and permeability barrier. After cell division, the daughter cells remain together and form a characteristic V-shape (Thanky *et al*., 2007; Letek *et al*., 2008). These cell cluster and aggregates most likely also contribute to the observed increase in FSC and DAPI signals in log phase populations where V-cells mainly occur (Fig. 2).

Heterogeneity with respect to cell size distribution was also described in previous microscopy-based studies of *C. glutamicum* (Gayen and Venkatesh, 2008; Grünberger *et al*., 2012). In the study of Gayen and Venkatesh the authors reported a difference in the dividing properties of small and large cells of this species. Cells with a surface area greater than 1.6 μm^2^ were categorized as maximally dividing cells; smaller cells exhibited significantly reduced proliferation capacity. A correlation between cell size and growth phase was also observed in a picolitre bioreactor microfluidic device were cells of *C. glutamicum* showed a size of ≥ 1.3 μm during exponential growth and a size of < 1.3 μm under carbon source limiting conditions (Gayen and Venkatesh, 2008; Grünberger *et al*., 2012). In general, reduction in cell size (in FSC signal, respectively) under carbon source limiting or stressful conditions has been described for various species (Erlebach *et al*., 2000; Hewitt and Nebe-von-Caron, 2004; Müller and Lösche, 2004; Díaz *et al*., 2010). For example, a significant decline in FSC values was reported for *S. cerevisiae* alcoholic fermentations, which was in fact associated with a reduction of actively proliferating yeast cells (Díaz *et al*., 2010). Furthermore, bacteria typically show a clear correlation between cell size and chromosome equivalents in the proliferation phase (Müller *et al*., 2010) – a fact which is also supported by the present study for *C. glutamicum* (Fig. 3). Thus, the measurement of the FSC and SSC distributions of cells in a bioreactor already provides valuable and easy accessible information on the overall fitness and metabolic activity of the bacterial culture.

### DNA pattern of *C. glutamicum*

**Figure 4:**
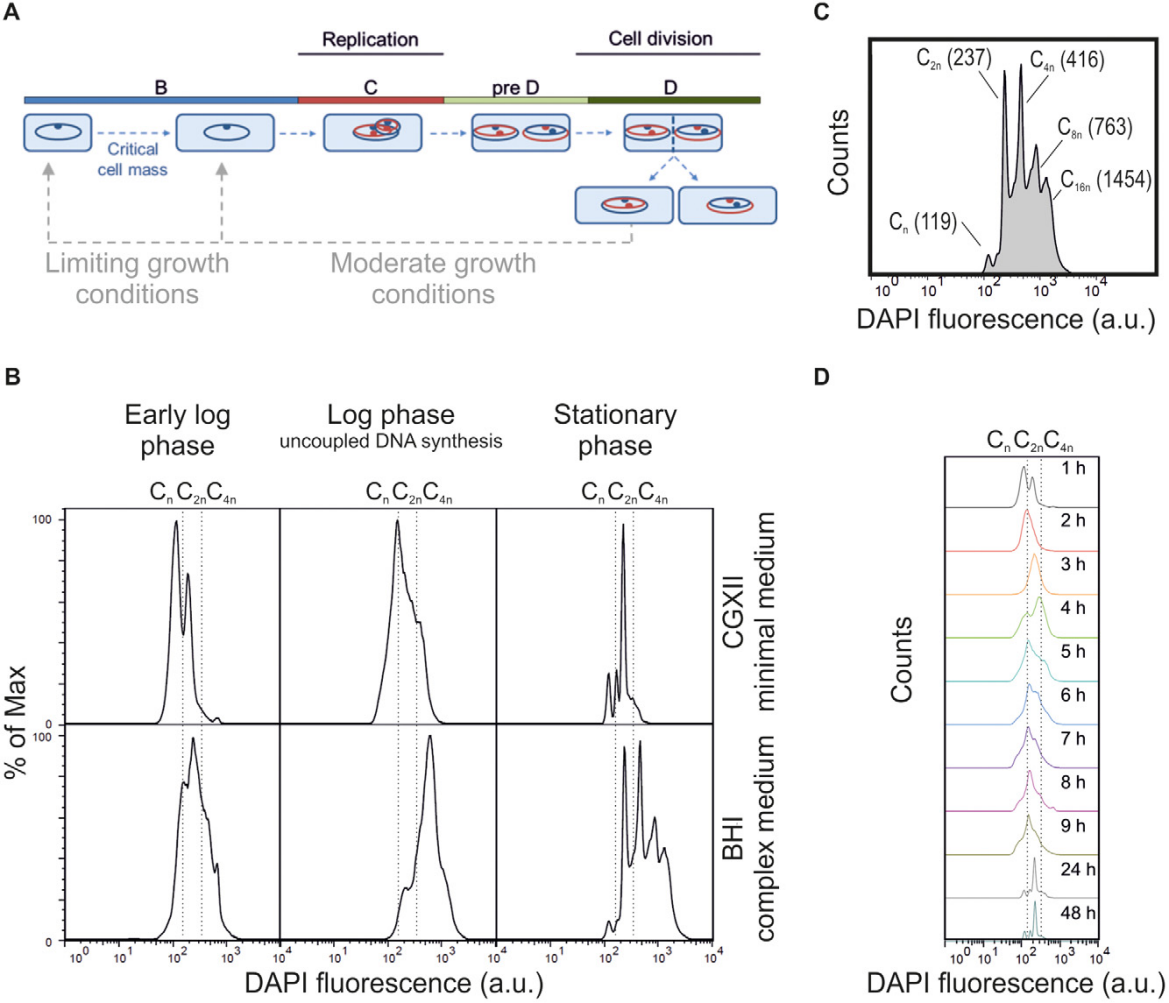
DNA patterns of *C. glutamicum* cells grown on minimal or complex medium.A. Schematic overview on the uncoupled cell cycle of bacteria (adapted from Müller *et al*., 2010). Within the B-phase cells increase their biomass before they are able to enter the C-phase. The duration of the B-phase depends on the environmental conditions. In the C-phase the DNA is replicated and chromosomes are segregated to the cell poles. The duration of the C-phase is species-dependent. When growth conditions are limiting, some species may remain in a so-called pre-D. The D-phase represents the phase of actual cell division.B. DNA patterns of *C. glutamicum* cells of the early logarithmic (log), log and stationary phase. Cells were cultured in CGXII minimal medium with 4% glucose or in BHI complex medium. After sampling, cells were fixed with 10% sodium azide (in PBS) and stained with DAPI (see *Experimental procedures*).C. Detailed view on DAPI histogram of stationary phase *C. glutamicum* cells (pre-D phase) grown on BHI complex medium. Indicated peaks correspond to different numbers of chromosome equivalents (C_n_).D. Time-course measurements of *C. glutamicum* cells during growth on CGXII minimal medium with 4% glucose.

In several recent studies the analysis of the DNA pattern of microbial populations has been proven as excellent tool for the determination of proliferation and cell cycle activities of microorganisms (Müller, 2007). In this study, we used DAPI staining to record the DNA pattern of *C. glutamicum* grown on CGXII minimal medium or BHI (brain heart infusion) complex medium in shake flasks. Samples were taken as indicated, DAPI stained and analysed in a flow cytometer equipped with a 355 nm solid state laser (see *Experimental procedures*). The strain *C. glutamicum* ATCC 13032 is known to contain one circular chromosome (Kalinowski *et al*., 2003). Since an absolute quantification of chromosome copy numbers requires more elaborated measurements, the first peak in a DAPI histogram is typically regarded as benchmark population C_n_, where C stands for chromosome equivalent and _n_ for the number thereof. Figure 4B shows the characteristic medium-dependent pattern that was observed for *C. glutamicum* cells grown on minimal or complex medium. Cells cultured in CGXII minimal medium typically showed two distinct peaks (C_n_ and C_2n_) in DAPI fluorescence at the early log phase, 1 h after inoculation into fresh medium. In the mid log phase, where *C. glutamicum* typically exhibits maximal growth rates, uncoupled DNA synthesis was observed as indicated by the shift of the population towards > C_2n_ chromosome equivalents. In the late log phase a backshift occurred and cells arrested within distinct C_Xn_ peaks in the stationary phase. On complex medium a similar tendency was observed, however, in comparison to minimal medium the distribution of DAPI peaks shifted towards higher C_Xn_ equivalents suggesting the occurrence of cells containing elevated numbers of chromosomes (Fig. 4B). This finding is in line with even better growth conditions on BHI medium resulting in higher growth rates of *C. glutamicum* (μ ∼ 0.6^−h^) compared with CGXII minimal medium (μ ∼ 0.4^−h^). In the stationary phase, again, the formation of distinct C_Xn_ peaks was detected with a maximum of chromosome equivalents of about C_16n_ (versus max. C_4n_ in CGXII).

The formation of distinct peaks within the late log and stationary phase can be explained due to the fact that bacteria finish replication (C-phase), but do not continue throughout cell division (D-phase) (Fig. 4A). Thus, our data reveal that *C. glutamicum* arrests in a pre-division (pre-D) phase when growth conditions become limiting. The occurrence of a pre-D phase under substrate limiting conditions has already been reported for several species, such as *Rhodococcus erythropolis* or *Acinetobacter* species, and was furthermore shown to readily pass over into cell division phase when growth conditions improve (Goodfellow *et al*., 1998; Müller *et al*., 2002). This is in agreement with the immediate entrance into D-phase when *C. glutamicum* stationary cultures were inoculated into fresh medium (see Fig. 4D, 1 h versus 48 h). Remarkably, previous studies revealed that occurrence of the pre-D phase is in fact a species-specific characteristic (Müller, 2007). The analysis of DNA patterns furthermore supports a symmetrical cell division of *C. glutamicum*, typical for rod-shaped species. We identified distinct peaks in the histogram corresponding to C_n_ (119), C_2n_ (237), C_4n_ (416), C_8n_ (763), C_16n_ (1454) etc. (Fig. 4) (Müller, 2007). However, we also observed minor peaks of odd numbers of chromosome equivalents, which is in fact indicative for a small fraction of asymmetrically dividing cells.

Altogether, these data highlight the value of DNA pattern analysis as rapid online-compatible tool for process monitoring and provide a first reference for *C. glutamicum* in standard minimal and complex media. Analysis of DNA patterns for the estimation of growth and activity states may also serve as a convenient alternative in turbid (e.g. CaCO_3_-buffered) production media which hamper the measurement of biomass as optical density or cell dry weight.

### FC-based measurement of the membrane potential

The MP plays an important role in the physiology of bacteria as it is intimately involved in the generation of ATP and has been implicated in bacterial autolysis, glucose transport, chemotaxis or bacterial survival at low pH. Therefore, the MP is a further adequate parameter to evaluate the cellular activity and metabolic state of a microbial population. Here, we used the carbocyanine dye 3,3′-diethyloxacarbocyanine iodide (DiOC_2_(3)) that exhibits a green fluorescence in all bacteria and shifts towards red emission due to self-association of dye molecules in dependency of the MP (high MP → increased red fluorescence) (Novo *et al*., 1999; 2000).

**Figure 5:**
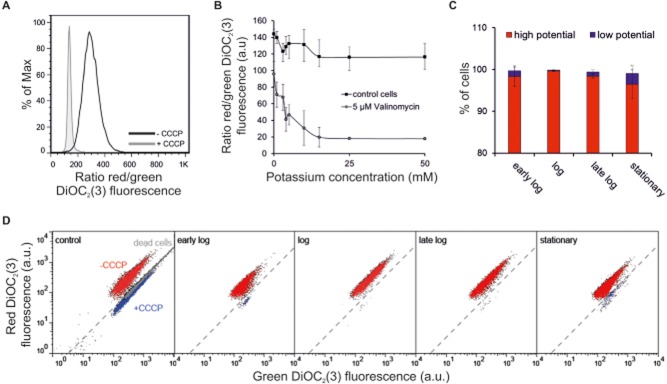
Determination of the MP of *C. glutamicum* cells.A. Control experiment ± 50 μM CCCP.B. Control experiments ± 5 μM valinomycin and different external potassium concentrations.C. Percentage of cells with a high or low MP. High and low MP were defined by the gating of the ± CCCP control shown in D. Bars represent the mean ± SD of three independent experiments.D. Representative dot plots of *C. glutamicum* cells of different growth phases (green DiOC_2_(3) fluorescence against the red DiOC_2_(3) fluorescence).

In a first set of experiments we verified suitability of DiOC_2_(3) for measurements in *C. glutamicum*. As a control, *C. glutamicum* cells were incubated with the uncoupler carbonyl cyanide *m*-chlorophenylhydrazone (CCCP), an inhibitor of oxidative phosphorylation, to a final concentration of 50 μM. CCCP-treated cells showed a considerable decrease in the red/green ratio of DiOC_2_(3) fluorescence indicative for a collapsed MP (Fig. 5A). For further validation we used the potassium ionophore valinomycin, which facilitates transport of potassium ions across the cell membrane. As a matter of fact, bacteria are depolarized when the external potassium concentration exceeds the cytoplasmic concentration (Novo *et al*., 2000). As expected, *C. glutamicum* log phase cells treated with 5 μM valinomycin showed a decrease in the red/green ratio of DiOC_2_(3) fluorescence in response to increasing external potassium concentrations. The MP collapsed rapidly in presence of potassium concentrations between 1 and 10 mM. A constant ratio was maintained for potassium concentrations > 15 mM (Fig. 5B).

Next, we analysed DiOC_2_(3) stained *C. glutamicum* cells grown in CGXII medium at different growth phases. Especially in early log and stationary phase populations we observed the formation of a second ‘low potential’ population, which resides at the position of CCCP-treated or dead cells (Fig. 5C and D). In the mid log phase, where cells grow at maximum rate, all cells showed vital states and almost no ‘low potential’ cells were detected. However, it has to be considered that a direct conclusion on cellular viability cannot be derived from FC measurements using fluorescent dyes as all dyes itself affect viability of treated cells (Table 1) (Nebe-von-Caron *et al*., 2000). We therefore rather emphasize MP measurements either for direct comparisons of different strains of the same species under well-defined conditions or as indirect tool to assess cellular viability during cultivation of a particular species.

**Table 1:** Survival rate of log and stationary phase *C. glutamicum* cells after treatment with different fluorescence dyes

	Survival rates (%)^a^							
	Unstained control		SYTO®9		DiOC_2_(3)		Propidium iodide	
Incubation time (h)	BHI	CGXII	BHI	CGXII	BHI	CGXII	BHI	CGXII
Log phase	96 ± 2	95 ± 4	80 ± 13	71 ± 34	< 1	< 1	< 1	< 1
Stat phase	63 ± 3	49 ± 4	66 ± 15	59 ± 29	< 1	< 1	< 1	< 1

a Data represent the percentage of visible colonies of sorted single cells of three independent experiments ± SD. Stained cells were considered as ‘dead’ when the survival rate was below 1%.

### Application of Syto 9 and propidium iodide – ‘live/dead’ staining

**Figure 6:**
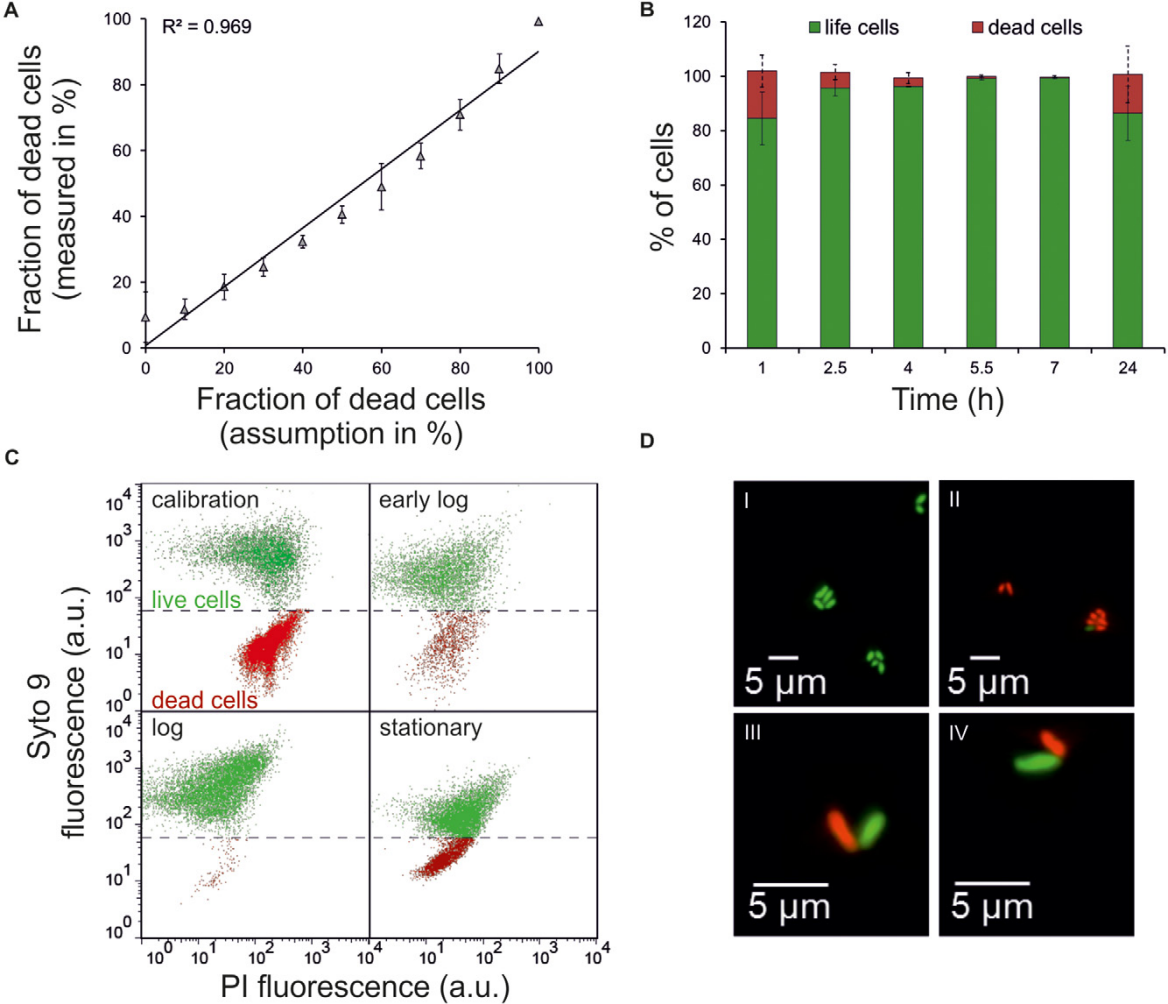
Live/dead staining of *C. glutamicum* cells using the fluorescent dyes PI and Syto 9. *C. glutamicum* ATCC 13032 was grown on CGXII minimal medium with 4% glucose, sampled as indicated and stained with PI (red) and Syto 9 (green) as described in *Experimental procedures*.A. Calibration: The assumed fraction of dead (isopropanol-killed) cells was plotted against the measured fraction of dead cells.B. Percentage of Syto 9 and Syto 9/PI positive (live) and PI positive (dead) cells in dependency of cultivation time. Bars represent the mean ± SD of three independent experiments.C. Dot plots (PI versus Syto 9) of the control experiment (calibration), early log, log, and stationary phase *C. glutamicum* cells (live: green; dead: red).D. Microscopic analysis (fluorescence channel) of log phase *C. glutamicum* cells (5.5 h after inoculation) stained with Syto 9 and PI. The majority of cells showed a green fluorescence (I), a small amount of cells stained PI positive (II). Often V-shaped sister cells were observed where one cell stained Syto 9 positive whereas the sister cell stained PI positive (III/IV).

A classical approach for the determination of membrane integrity is the combination of the fluorescent dyes propidium iodide (PI) and Syto 9, known as ‘live/dead staining’ or BacLight (Hammes *et al*., 2008). Both dyes bind specifically to DNA and RNA by intercalation between bases. Whereas Syto 9 (green fluorescence) is membrane permeable and stains all cells, PI only penetrates cells with damaged membranes and masks in these cells the Syto 9 signal. For verification of the staining protocol we performed a calibration using defined mixtures of live and dead (isopropanol-killed) cells stained with PI and Syto 9. FC analysis of these mixtures revealed significant agreement of measured and assumed cell fractions (Fig. 5A). Application of this dye combination on *C. glutamicum* cells of different growth phases resulted in the identification of a PI positive subpopulation in the early log phase, which significantly declines in the log phase and reappears in the stationary phase (Fig. 6B and C). This increase in PI positive cells correlated well with the distribution of a low MP population (Fig. 5). However, absolute values differ between the applied staining approaches and again highlight that these approaches are not suitable for the exact counting of live and dead cells in a population, but rather show a characteristic pattern in a particular growth phase.

Remarkably, we also observed a shift of log phase cells towards a higher PI and Syto 9 signal (Fig. 6C). This behaviour was also described for several other species and is supposed to be due to loosen cell wall structures during periods of fast cell size growth and high division rates. However, the actual reason why the cell walls of some dividing cells might become permeable for PI remains hitherto unknown (Lybarger and Maddock, 2001; Shi *et al*., 2004). Interestingly, in microscopic analysis we often observed (besides green or red cells, Fig. 6D I/II) V-shape cells where one cell stained green and the other one red (Fig. 6D III/IV). This phenomenon was observed for more than 50% of V-shape cells in the exponential phase and for about 33% in the stationary phase. This finding has also been described for *Corynebacterium diphtheria* (Ott *et al*., 2010), but the reason for this staining pattern is so far unknown. However, it is very unlikely that red cells of V-shapes, occurring during fast cell division in log phase populations, represent ‘dead’ *C. glutamicum* cells. It can rather be suggested that this phenomenon is caused by the specific corynebacterial cell wall composition and cell division resulting in one PI impermeable and one permeable sister cell.

### Conclusion

In the present study we have applied FC as rapid and convenient tool to evaluate growth behaviour and viability of *C. glutamicum* at different growth stages. The reported FC approaches combine the advantage of rapid, online-compatible measurements with high-throughput analysis at single cell resolution. The large repertoire of available stains provides access to a variety of cellular parameters and can be combined in multiparameter FC analysis. Recent efforts of several groups aim on the development of downstream analytics, such as transcriptomics or proteomics approaches, which can be applied for the further analysis of FC sorted subpopulations to obtain insights into phenotypic variation at the molecular level (Bernas *et al*., 2006; Wiacek *et al*., 2006; Jehmlich *et al*., 2010). Recently, our group also reported the development of a genetically encoded single cell biosensor for the intracellular detection of l-methionine and branched-chain amino acids in *C. glutamicum* (Mustafi *et al*., 2012). Application of this sensor in the l-valine-producing *C. glutamicum* strain Δ*aceE* already suggested significant variation with respect to amino acid production at the single cell level. In this context, the application of multiparameter cytometry, making use of genetical biosensors and/or fluorescent dyes, will provide more insights into phenotypic variation of industrial production strains and the underlying mechanisms thereof.

Our study, as well as previous ones with other model species, highlights the importance of a thorough verification of available fluorescent dyes and the adaptation of the respective staining protocols for each particular species of interest. Overcoming this obstacle, muliparameter cytometry provides an efficient online tool to rapidly monitor growth states and metabolic activity of microbial populations in bioprocesses.

## Experimental procedures

### Bacterial strains, media and growth conditions

In the following experiments *C. glutamicum* ATCC 13032 was used as wild type strain (Kalinowski *et al*., 2003). A first pre-culture of *C. glutamicum* was inoculated with colonies from a fresh BHI agar plate (brain heart infusion, Difco™ BHI, BD, Heidelberg, Germany) and grown in 5 ml BHI liquid medium for 8 h at a temperature of 30°C and a shaking rate of 170 r.p.m. Afterwards, cells were washed with 0.9% (w/v) NaCl and were transferred to 50 ml CGXII minimal medium with 4% (w/v) glucose (Keilhauer *et al*., 1993). Cells were cultured overnight at 30°C with a shaking rate of 120 r.p.m. For the main culture, CGXII medium was inoculated to an OD_600_ of 1 and *C. glutamicum* was grown at 30°C for 48 h in 500 ml shaking flasks in 50 ml medium. The growth of *C. glutamicum* in CGXII medium with 4% glucose or BHI complex medium is shown in Fig. 1.

### Staining of bacteria

Samples of *C. glutamicum* were harvested from 50 ml cultures at different time points as indicated. OD_600_ was measured and the samples were diluted in phosphate-buffered saline (PBS) (129 mM NaCl, 2.5 mM KCl, 7 mM Na_2_HPO_4_ × 2H_2_O, 1.3 mM KH_2_PO_4_, pH 7.0) or FACSFlow™ sheat fluid buffer (BD, Heidelberg, Germany). Afterwards, cells were stained with the different fluorescence dyes as described below. Table 2 provides an overview on used dye concentrations and incubation times.

**Table 2:** Filter set-ups, concentrations, incubation times and function of used fluorescence dyes

Fluorescence dye	Excitation/emission (nm)	Long-pass filter (nm)	Band-pass filter (nm)	Concentration (μM)	Incubation time (min)	Function
DAPI^a^	355/463	–	450/65	0.25	60	DNA dye
DiOC_2_(3)				30	30	Membrane potential
Green fluorescence	488/497	502	530/30			
Red fluorescence	488/610	565	610/20			
PI	538/617	595	610/20	20	15	Nucleic acid dye/membrane integrity
Syto9	485/498	502	530/30	3.3	15	Nucleic acid dye/cell count

a Analyses were performed with a MoFlo cytometer (see *Experimental procedures*).

### DNA patterns

Bacteria were harvested by centrifugation (4.000 *g*, 5 min, 4°C) and washed in phosphate buffer (0.4 M Na_2_HPO_4_/NaH_2_PO_4_, 150 mM NaCl, pH 7.2). Subsequently, cells (diluted to an OD_600_ of 1) were fixed with phosphate buffer containing 10% sodium azide and stored at 4°C until cytometric measurement for at least 1 h and no longer than 1 week. Two ml of the cell suspension (diluted to OD_600_ = 0.035) were treated with 1 ml solution A (0.1 M citric acid and 4.1 mM Tween 20) for 10 min at room temperature (RT). Afterwards, cells were stained with 0.25 μM DAPI (Sigma-Aldrich, Taufkirchen, Germany) in 0.4 M sodium phosphate solution (Na_2_HPO_4_) pH 7.0 for 60 min at RT in the dark using a modification of a standard procedure (Meistrich *et al*., 1978).

### Membrane potential

*C. glutamicum* cells were diluted to an OD_600_ of 0.05 and stained with 30 μM DiOC_2_(3) [(stock solution: 3 mM in dimethylsulphoxide (DMSO)] (Sigma-Aldrich) for 30 min at RT. We chose this long incubation time, since the DiOC_2_(3) signal is shifting rapidly during the first 30 min towards higher fluorescence intensities. This would require a to the split second measurement to achieve reproducible results. An experiment where cells were stained for only 1 min showed a similar pattern/as cells stained for 30 min (data not shown). For CCCP (carbonyl cyanide *m*-chlorophenylhydrazone) control experiments, cells were incubated with 50 μM CCCP (stock solution 5 mM in DMSO) (Sigma-Aldrich). In a further proof of principle experiment, cells were treated with 5 μM of the potassium ionophore valinomycin (stock solution 5 mM in DMSO) (Sigma-Aldrich) and stained in phosphate buffer (pH 7) containing different concentrations of KCl (0–300 mM) and appropriate amounts of NaCl (300-x, x = KCl concentration) (Novo *et al*., 2000).

### Live-dead staining

Cells with an OD_600_ of 0.05 were incubated with 20 μM propidium iodide (PI stock solution: 20 mM in DMSO) (Molecular Probes, Leiden, Netherlands) and 3.3 μM Syto 9 (stock solution: 3.34 mM in DMSO) (Molecular Probes, Leiden, Netherlands) for 15 min at RT. For validation of the protocol, intact cells and cells with injured membranes, (treated with 70% isopropyl alcohol for 30 min) were mixed in different ratios. Afterwards, cells were stained as described above.

### Flow cytometry

Flow cytometry analyses and cell sorting experiments were performed with a FACSAria II flow cytometer (Becton Dickinson, Heidelberg, Germany) using a blue solid state laser (Sapphire™ 488-20) with an excitation wavelength of 488 nm and a power of 13 mW. Cytometer set-up and performance tracking was performed with Cytometer Setup 0026; Tracking Beads [bright (3 μm), mid (3 μm), and dim (2 μm) beads] labelled with a mixture of fluorochromes (Becton Dickinson).

DNA analyses were performed on a MoFlo (DakoCytomation, FortCollins, CO) equipped with a water-cooled argon-ion laser (Innova 70C from Coherent, Santa Clara, California, USA). Excitation of 400 mW at 488 nm was used to analyse the FSC and SSC as trigger signal at the first observation point. DAPI was excited by 150 mW with a 355 nm solid state laser XCyte (JDS Uniphase) at the second observation point. The orthogonal signal was first reflected by a beamsplitter and then recorded after reflection by a 555 nm long-pass dichroic mirror, passage by a 505 nm short-pass dichroic mirror and a BP 488/10. DAPI fluorescence was monitored using a 450/65 band pass filter. Amplification was carried out at logarithmic scales. Fluorescent beads (0.5 μm bright blue Fluoresbrite carboxylate microspheres, 360/407, Polyscience, Warrington, Pennsylvania, USA) were used to align the MoFlo. Also, an internal DAPI stained bacterial cell standard was introduced for tuning the device up to a CV value not higher than 6%. Table 2 summarizes filter set-ups used for the analysis of the different fluorescence dyes. Ten thousand cells per sample (40 000 cells for DAPI measurements) were recorded using FACSDiva software 6.0 (FACSAria II) or Summit v4.3 software (MoFlo). Data were analysed with the FlowJo FC analysis software 7.6.5 (Tree Star, Ashland, USA).

### Survival rate

For determination of the cellular survival rate after different staining procedures single cells were sorted with the four-way-purity precision mode onto BHI or CGXII agar plates (256 single cells per agar plate). Plates were incubated at a temperature of 30°C for 48 h. Afterwards, the percentage of visible colonies of sorted single cells was determined and was regarded as survival rate (Table 1).

### Statistical analysis

Data are expressed as mean ± standard deviation (SD) of three independent biological replicates. Statistical significance was calculated via *t*-test; *P*-values ≤ 0.05 were considered to be statistically significant.
